# A Cure for Consumption

**Published:** 1887-06

**Authors:** 


### A CURE FOR CONSUMPTION.

It will be welcome news to thousands that, what is claimed to be a cure for that most fatal disease, *consumption*, has at last been discovered and applied with most satisfactory results.

It was in 1883, that M. Debove, professor at the "*Hopital de la Pitie*, Paris, asserted in a lecture that Consumption, being due to the presence of a parasite, the proper treatment of it was by the use of a parasiticide. Acting upon this suggestion, M. Bergeon of the Lyon's Medical School, put it in actual practice, by injecting into the system of consumptive patients by natural channnels, sulphurretted hydrogen, carrying twenty-five parts by weight of tartaric acid, and one part by weight of solicylic acid. The success of this treatment is admitted to be remarkable. The cough is at once allayed ; the expectoration diminishes or ceases altogether ; the lost appetite is regained, and sleep, natural and undisturbed ; in short, this terrible disease which has so long defied the skill of the ablest physicians, has at length been fairly conquered.

In this country the treatment above indicated sometimes slightly varied, has been adopted in a large number of cases at the Philadelphia Hospital, with equally promising results.

It is also claimed that it is equally efficatious in cases of asthma and chronic catarrh.

